# The Continued Rise of Syphilis: A Case Report to Aid in Identification of the Great Imitator

**DOI:** 10.21980/J8KM02

**Published:** 2023-04-30

**Authors:** Nicole Finney, Eli Soyfer, Rory Schwan, Lindsey C Spiegelman

**Affiliations:** * University of California, Irvine, Department of Emergency Medicine, Orange, CA

## Abstract

Syphilis is a progressive sexually transmitted infection that has a wide variety of presentations depending on the disease stage. These variable presentations can make it difficult to differentiate syphilis from other diseases. While tertiary syphilis is less common in the United States compared to primary or secondary syphilis, recognition of the varied manifestations of advanced syphilis can help providers accurately diagnose this disease to help prevent continued spread. In this case report, we present a patient with a history of bilateral palmar wounds. The patient had presented to multiple emergency departments without a diagnosis of syphilis. Upon subsequent emergency department visits, the patient was diagnosed with tertiary syphilis, started on a course of penicillin, and evaluated by dermatology. However, the patient left against medical advice prior to further evaluation and treatment. From this case report, we have learned the importance of considering tertiary syphilis gummas in the differential diagnosis of atypical skin wounds.

**Topics:**

Sexually transmitted infection (sti), syphilis, tertiary syphilis, gummas, dermatologic lesions.

## Brief introduction

While rare, tertiary syphilis should be included on the differential diagnosis of skin wounds given its nonspecific manifestations. Syphilis is a sextually transmitted infection (STI) caused by the bacteria *Treponema pallidum*. As with other STIs, individuals who engage in high-risk behaviors, such as use of intravenous (IV) drugs, multiple sexual partners, and unprotected sex, are at an increased risk of contracting syphilis. However, while known for its sexual transmission, syphilis can be transmitted via any contact with infected bodily fluids through mucus membranes or compromised skin. This disease progresses through three stages, each with its own clinical presentation: primary (chancres), secondary (maculopapular or pustular rash, nonspecific flu-like symptoms), latent (asymptomatic period with serologic proof of infection), and tertiary (gummas, neurological and cardiovascular involvement).^1^ The variability of symptoms across disease progression and similarities to other diseases can lead to a broad list of differential diagnoses complicating the accurate diagnosis of syphilis.^2^,^3^ For this reason, providers need to have a high index of suspicion for syphilitic sequelae. In this review, we show how diagnosing tertiary syphilis can be complicated by incomplete patient histories, partial treatment at outside hospitals, and the overlap of tertiary syphilis symptoms with other diseases. Written informed consent was obtained for patient photographs and publication.[Fig f1-jetem-8-2-v11][Fig f2-jetem-8-2-v11]

**Figure f1-jetem-8-2-v11:**
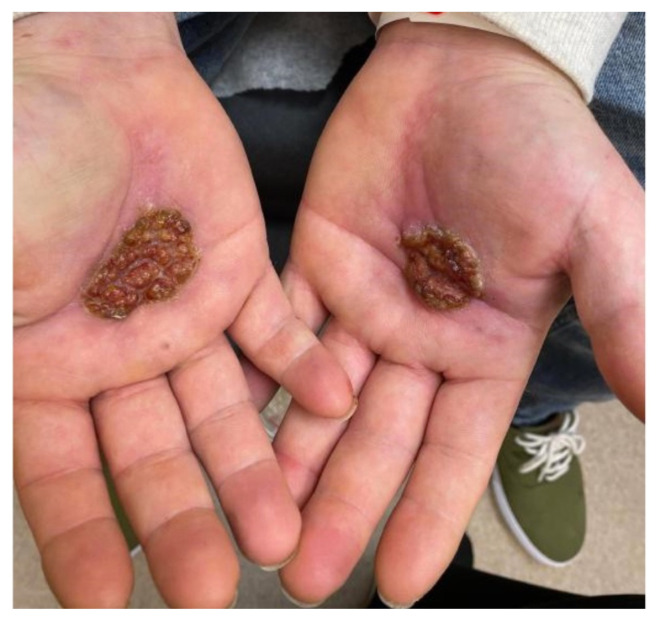


**Figure f2-jetem-8-2-v11:**
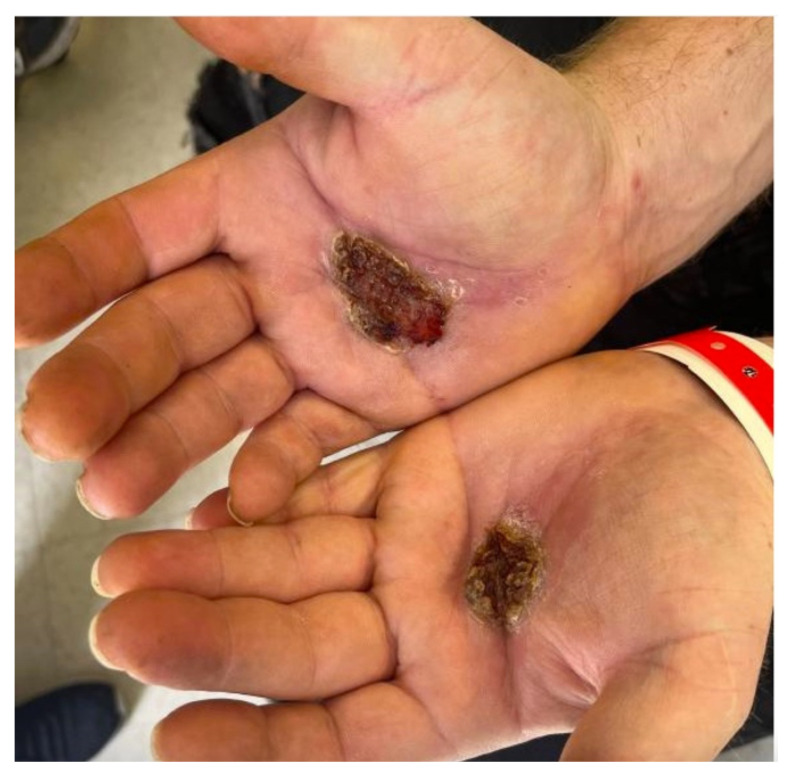


## Presenting concerns and clinical findings

A 36-year-old male with no known past medical history presented to the emergency department (ED) with wounds to the palmar aspects of his bilateral hands for three months. The wounds began as small blisters after a reported chemical spill and progressively worsened. For two months after the injury, the patient applied hydrogen peroxide daily to his wounds. During this time, the patient visited an outside ED twice for a wound check. At the first visit, he was diagnosed with a chemical burn and skin infection and discharged with clindamycin, lactobacillus probiotic products, and mupirocin 2% ointment. The patient returned for a second visit, stating he took the medications as prescribed, but his hand wounds were now painful with a serous discharge. He was given 1 gram of ceftriaxone intravenously and discharged with doxycycline and hydrocodone-acetaminophen. The following day he presented to our ED. He reported significant sharp pain in his palms, even waking him from sleep, which was improved by submerging his hands in cold water. To our providers, the patient disclosed polysubstance use (methylphenidate, fentanyl, methamphetamine, marijuana, and cocaine) and tobacco use. He denied any distal numbness or tingling but reported intermittent subjective fevers. He also denied any penile lesions but endorsed previous lesions on his thighs. Examination of his bilateral palms showed ulcerating, purulent wounds with a foul odor that were exquisitely tender to palpation.

## Significant findings

Images taken of the bilateral palmar skin lesions at our institution showed multi-centimeter, well-demarcated, friable, verrucous, crusted plaques with overlying fine yellow crust. Lesions such as these are suspicious for syphilitic gummas seen with cutaneous tertiary syphilis.

## Patient course

While in our ED, dermatology was consulted. Tissue samples were taken for acid-fast bacillus (AFB), bacterial, and fungal culture, and a punch biopsy for dermatopathy was performed at the bedside. The patient revealed he was sexually active with female partners and inconsistently used condoms. He further disclosed that the lesions first erupted one week after being in a hot tub with a partner. On his first visit to our institution, labs were notable for borderline erythrocyte sedimentation rate (ESR) elevation, normal electrolytes, normal blood urea nitrogen (BUN)/creatinine (Cr) ratio, normal liver function tests (LFTs), negative lactic acid, no leukocytosis, borderline anemia, and human immunodeficiency virus (HIV) nonreactive. Blood cultures were sent. A syphilis rapid plasma reagin (RPR), hepatitis B/C serologies, and urine gonorrhea/chlamydia (GC/CT) were sent for infection and STI work-up. The patient was cleared by dermatology for discharge with doxycycline for infection and hydrocodone-acetaminophen for pain control.

The next day, the patient was called back to our ED for further evaluation and treatment after labs resulted with a positive RPR with a titer of 256. After being informed of his syphilis diagnosis, he returned to our ED. At this time, the patient reported concern that his rash was from a hot tub exposure or a contact with known syphilis wiping a dirty rag on his hands. The patient confirmed an extensive history of drug use, including intravenous drugs. He also newly reported periods of confusion, which he attributed to his drug use, and blurry vision and subjective hand weakness for the past year. Beyond his bilateral hand lesions, physical exam in the ED, including neurological testing, was non-contributory. Additional labs were notable for a positive confirmatory *Treponema palladium* test. During his second ED visit at our institution, infectious disease was consulted, and they recommended a lumbar puncture if neurological symptoms presented and admission to internal medicine. The patient was given penicillin G 2.4 million units and admitted for inpatient management.

After being admitted to medicine for further work up and continued penicillin G treatments, a genitourinary exam was performed. On physical exam, the medicine team noted two erythematous lesions on the ventral penis measuring less than one centimeter, two erythematous lesions on his left scrotum, one with a “mounded up” white center on an erythematous base. STI testing was negative for gonorrhea, chlamydia, trichomonas, HIV, hepatitis C, and hepatitis A. However, the patient had a positive reaction for hepatitis B surface antigen, and it was determined that he was immune to hepatitis B infection. The infectious disease team recommended repeating the RPR to monitor the patient’s response to treatment. The dermatology team noted the hand lesions as being “very friable fungating crusted plaques.” Their biopsy of the patient’s lesions revealed spirochetes within the fibrinous dermal tissue, confirming the diagnosis. Gram staining highlighted gramnegative cocci within the fibrinous dermal tissue. Fite stain for leprosy and Grocott’s methenamine silver (GMS) stain for fungi were negative.

Unfortunately, one day after admission, the patient left against medical advice prior to completing treatment and work-up. He refused to wait for medications to be sent to the pharmacy or any discharge paperwork. Prior to his leaving, nursing informed and encouraged the patient and his family to return or go to another hospital for further treatment. The lumbar puncture recommended by infectious disease was not able to be completed prior to the patient leaving against medical advice. Similarly, the medicine team was unable to consult ophthalmology for any potential vision changes. The department of public health was notified of the patient’s syphilis status upon his discharge.

## Discussion

In this case report, we discuss a patient whose gummatous hand lesions were initially inappropriately diagnosed as chemical burns with superimposed cellulitis. While the patient’s self-reported history supported a non-infectious etiology, clinical suspicion for and subsequent diagnosis of syphilis was only heightened after failure of initial burn treatment. We hope that this report and its associated images can help guide expansion of the differential diagnosis to include cutaneous presentation of gummatous disease.

With its constellation of nonspecific symptoms, syphilis has long earned itself the nickname, the Great Imitator.^3^ Tertiary syphilis is rare. However, the prevalence of syphilis in the United States has been increasing since 2001.^4^,^5^ For patients presenting with skin findings, particularly in the setting of intravenous drug use and high-risk sexual behaviors, it is important to keep syphilitic gummas in the differential, particularly if lesions are abnormal or resolution is not as anticipated.

While the venereal disease research laboratory (VDLR) screening test for syphilis is commonly included in STI panels, results fluctuate and remain negative in 25% of patients.^6^ About 30% of cases of untreated syphilis will eventually progress to tertiary syphilis, 16% of which will exhibit cutaneous syphilis.^7^ However, in the case of skin lesions of late secondary and tertiary syphilis, more definitive tests that visualize the spirochete, such as darkfield microscopy, often fail since organisms degrade with time.^8^,^9^ Progression to tertiary syphilis takes place between months to years but may be faster (within weeks) with HIV co-infection.^6^,^10^,^11^ Gummas, an inflammatory response to the syphilis spirochete, are a sequelae of tertiary syphilis that develop one to 46 years after infection, usually within the first 15 years.^7^,^10^

Cutaneous tertiary syphilis is further subdivided into a superficial noduloulcerative type affecting only the skin, and a deeper gummatous type, affecting skin, bone, and liver.^10^,^12^ The noduloulcerative type is more common, appearing as superficial, flat nodules or plaques that develop a serpiginous configuration resembling granuloma annulare, lupus vulgaris, psoriasis, or sarcoid.^2^,^13^ These cutaneous lesions are usually asymptomatic, but up to 42% of patients report pruritus.^13^

The deeper gummatous type presents as a non-tender nodular lesion with central punched-out necrosis, which can appear similar to other skin pathologies like cancer or a wound, as in our patient.^2^,^14^ Gummas are typically 2 – 10 cm large.^15^ The gummatous skin lesions may appear anywhere on the body and have been described on the face, oral cavity, and penis, as well as the hands, as in our patient.^2^,^10^,^13^,^14^,^16^ In particular, it is prudent to add tertiary syphilis to the short differential of skin rashes found on the palm and soles. If gummas affect the bone, imaging shows areas of bony lysis surrounded by dense sclerosis.^10^ Lastly, if affecting the internal organs, there will be a mass-life effect with CT showing masses with low attenuation, rarely with calification.^17^

Given the later stage of disease, tertiary syphilis and its cutaneous manifestations are treated with multiple, large doses of penicillin. Recommended treatment is 3 once-weekly benzathine penicillin G, 2.4 million units given intramuscularly.^10^,^14^ Good response to this treatment can help support the diagnosis of syphilitic gummas. For additional monitoring, quantitative nontreponemal test titers should decrease 4-fold within 12 to 24 months after treatment of latent or late syphilis.^18^

While the diagnosis of syphilis relies heavily on physical exam findings and laboratory results, as we saw with this patient, the social history also plays a particularly influential role. This history may become even more important in the case of tertiary syphilis where syphilitic lesions may be hard to distinguish from other pathologies. Fostering a trusting patient-provider relationship is key to making patients feel comfortable disclosing more vulnerable parts of their social history. Identifying patients who engage in high-risk behaviors may heighten clinicians’ suspicion for syphilis and increase testing. Additionally, expanding differentials for patients with continued symptoms and repeat ED visits may help identify and diagnose other cases of tertiary syphilis.

The varied appearance of cutaneous syphilis makes the diagnosis challenging. Given the recent rise of syphilis cases and the public health concern linked to spread, it is important to keep syphilis on the differential diagnosis of atypical skin wounds. Syphilis, particularly progression to tertiary syphilis, is still rare in the United States. Therefore, a broad differential of common skin pathologies should be investigated as well.

## Supplementary Information





## References

[1] Ivars Lleó M Clavo Escribano P Menéndez Prieto B Atypical cutaneous manifestations in syphilis Actas Dermo-Sifiliográficas (English Edition) 2016 107 4 275 283 10.1016/j.adengl.2016.02.002 26708562

[2] Moon J Yu DA Yoon HS Cho S Park HS Syphilitic Gumma: a rare form of cutaneous tertiary syphilis Ann Dermatol 2018 Dec 30 6 749 751 Epub 2018 Oct 26 10.5021/ad.2018.30.6.749 33911527 PMC7992438

[3] Rodriguez S Teich DL Weinman MD Greene JM Keroack MA Apstein MD Gummatous syphilis: a reminder The Journal of Infectious Diseases 1988 157 3 606 607 10.1093/infdis/157.3.606 3343534

[4] de Voux A Kidd S Torrone EA Reported cases of neurosyphilis among early syphilis cases—United States, 2009 to 2015 Sex Transm Dis 2018 45 1 39 41 10.1097/OLQ.0000000000000687 28876294 PMC5763486

[5] Preliminary 2021 STD Surveillance Data Centers for Disease Control and Prevention Published September 1, 2022 Accessed September 1, 2022 https://www.cdc.gov/std/statistics/2021/default.htm

[6] Rocha N Horta M Sanches M Lima O Massa A Syphilitic gumma – cutaneous tertiary syphilis J Eur Acad Dermatol Venereol 2004 18 4 517 518 10.1111/j.1468-3083.2004.00960.x 15196182

[7] Gurney Clark E Danbolt N The Oslo Study of the natural course of untreated syphilis: an epidemiologic investigation based on a re-study of the Boeck-Bruusgaard material Med Clin North AM 1964 48 3 613 623 10.1016/S0025-7125(16)33445-9 13252075

[8] Nayak S Acharjya B VDRL Test and its Interpretation Indian J Dermatol 2012 57 1 3 8 10.4103/0019-5154.92666 22470199 PMC3312652

[9] Zoechling N Schluepen EM Soyer HP Kerl H Volkenandt M Molecular detection of Treponema pallidum in secondary and tertiary syphilis Br J Dermatol 1997 136 5 683 686 9205499

[10] Masege SD Karstaedt A A rare case of a chronic syphilitic gumma in a man infected with human immunodeficiency virus JLO 2014 128 6 557 560 10.1017/S0022215114001200 24909596

[11] Boyd AS Syphilitic gumma arising in association with foreign material J Cutan Pathol 2016 43 11 1028 1030 10.1111/cup.12770 27427500

[12] Crowson N Magro C Mihm M Jr Treponemal diseases Elder D Elenitsas R Jaworsky C Johnson B Lever’s Histopathology of the Skin 8th ed Lippincott-Raven 1997 503 508

[13] Wu SJ Nguyen EQ Nielsen TA Pellegrini AE Nodular tertiary syphilis mimicking granuloma annulare J Am Acad Dermatol 2000 42 2, Part 2 378 380 10.1016/S0190-9622(00)90117-8 10640938

[14] Benzaquen M Horreau C Koeppel MC Berbis P A pseudotumoral facial mass revealing tertiary syphilis Clin Exp Dermatol 2017 42 6 714 716 10.1111/ced.13134 28543323

[15] Varejão AM Monteiro DM Peixinho C Perineal syphilitic gumma: tertiary syphilis in a developed country BMJ Case Reports CP 2022 15 6 e250564 10.1136/bcr-2022-250564 PMC9244684 35768162

[16] Asselin C Ekindi N Carignan A Richard PO Gummatous penile syphilis IDCases 2019 18 e00589 10.1016/j.idcr.2019.e00589 31406680 PMC6685700

[17] Shapiro MP Gale ME Tertiary syphilis of the liver: CT appearance Journal of Computer Assisted Tomography 1987 11 3 546 3571608 10.1097/00004728-198705000-00038

[18] Little JW Syphilis: An update Oral Surg Oral Med Oral Pathol Oral Radiol 2005 100 1 3 9 10.1016/j.tripleo.2005.03.006 15953910

