# Supplementary figures and images for: The Continued Rise of Syphilis: A Case Report to Aid in Identification of the Great Imitator
Source: J Educ Teach Emerg Med. 2023 Apr 30;8(2):V11–5. doi: 10.21980/J8KM02 (PMC10332677; doi:10.21980/J8KM02)

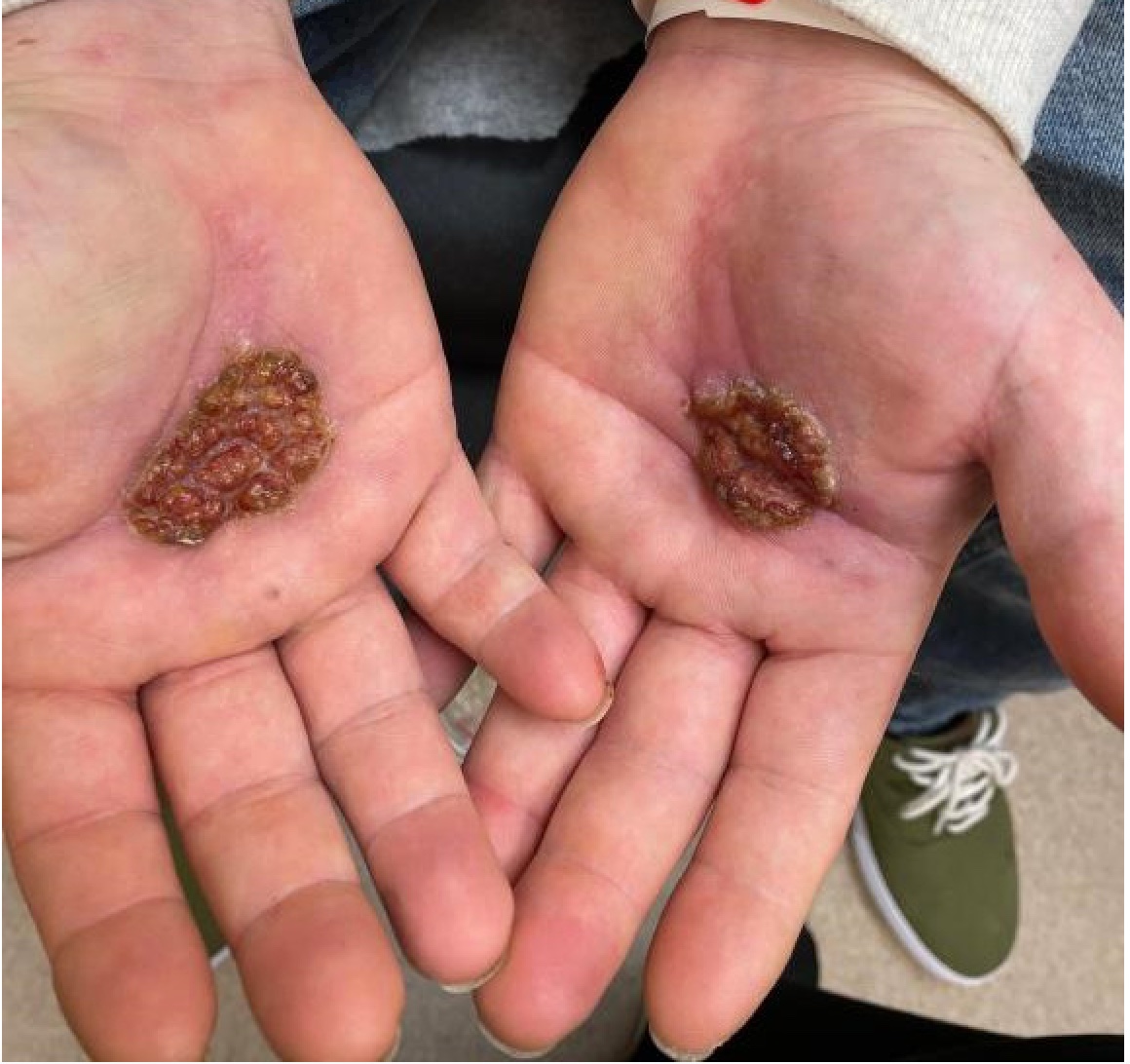

Supplement: Supplementary file 1 [file JETem-8-2-V11-supp1.jpg]

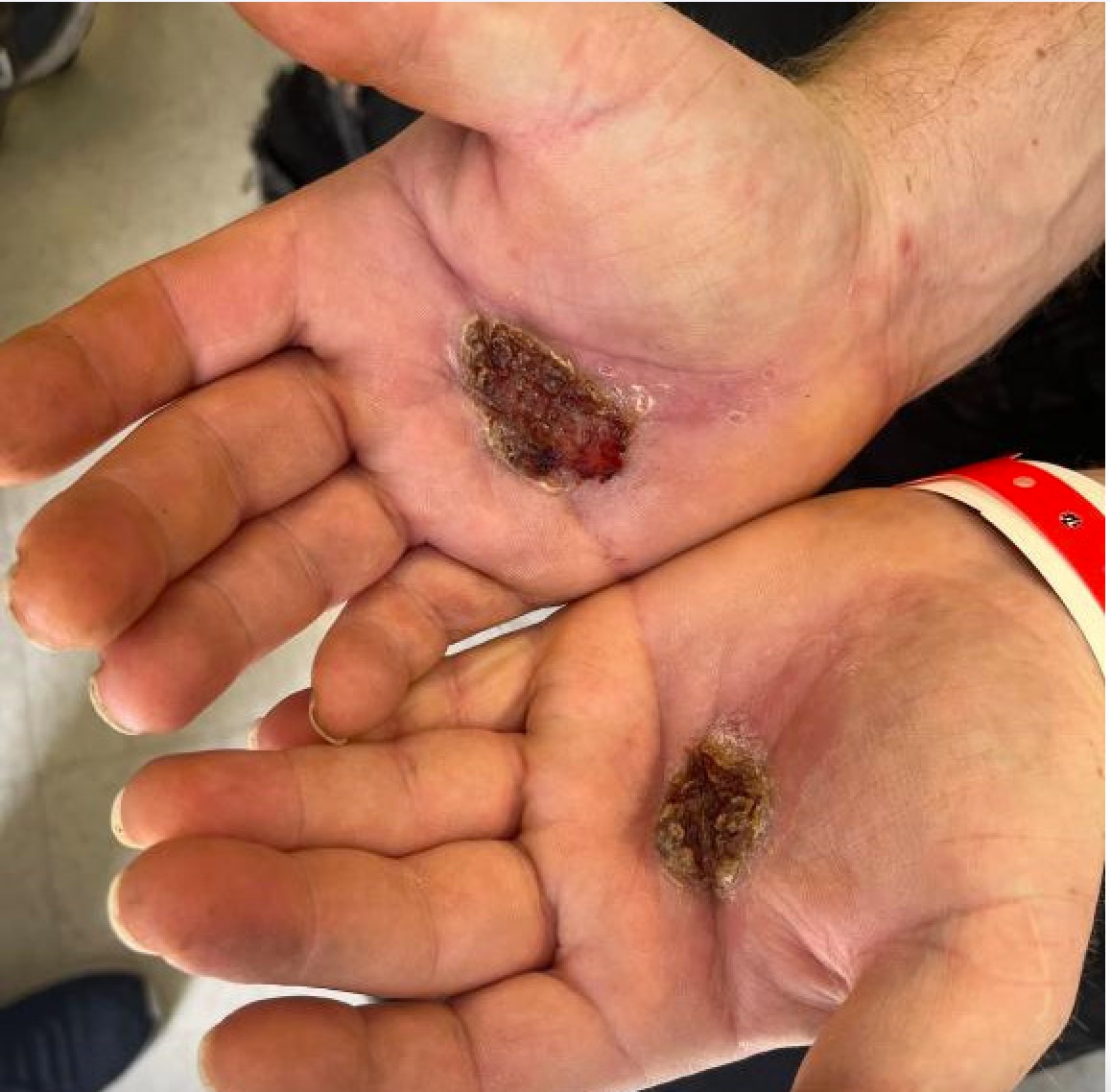

Supplement: Supplementary file 2 [file JETem-8-2-V11-supp2.jpg]
